# Correlation between internal pudendal artery stenosis and erectile dysfunction in patients with suspected coronary artery disease

**DOI:** 10.1371/journal.pone.0225179

**Published:** 2019-11-12

**Authors:** Ha-Wook Park, Sung-Ho Her, Bong-Hee Park, Dong-Seok Han, Seung Mo Yuk, Dae-Won Kim, Chang Shik Youn, Hoon Jang

**Affiliations:** 1 The Department of Cardiology, College of Medicine, The Catholic University of Korea, Seoul, Republic of Korea; 2 The Department of Urology, College of Medicine, The Catholic University of Korea, Seoul, Republic of Korea; Universidad Francisco de Vitoria, SPAIN

## Abstract

**Background:**

Stenoses of internal pudendal arteries (IPAs) appear to be related to erectile dysfunction (ED). Nevertheless, the correlation between the severity of ED and stenosis of the IPAs is not well established.

**Objectives:**

To evaluate angiographic findings of IPAs in patients with suspected coronary artery disease (CAD) and to assess the correlation between the severity of ED and IPA stenosis.

**Materials and methods:**

Ninety-one patients who were scheduled for cardiac angiogram (CAG) because of suspected CAD participated. ED was assessed using the International Index of Erectile Function (IIEF) questionnaire. Erectile function (EF) domain scoring was used to assess the severity of ED: severe (EF score = 1–10); moderate (11–16); mild-moderate (17–21); mild (22–25); and no ED (26–30). Angiography was performed in bilateral common, internal iliac, and IPAs and the location and extent of stenoses were measured. We divided patients according to those with maximum stenosis of less than 50% (Group I) and those with more than 50% (Group II), regardless of direction.

**Results:**

We diagnosed 88 patients (88/91, 96.70%) with ED. There was no correlation between increasing age and severity of ED (r = - 0.063, p = 0.555). There were 72 patients in Group I and 19 in Group II. In Group I, 62 patients were diagnosed with ED even though there was no stenosis. There was no significant correlation between the severity of ED and the extent of stenosis in IPAs (r = -0.118, p = 0.265).

**Conclusions:**

There was no significant correlation between the severity of ED and the extent of stenosis of IPAs. We believe that this is because the progression of ED is induced by endothelial cell dysfunction, not by mechanical obstruction leading to blood flow reduction.

## Introduction

Erectile dysfunction (ED), defined as the persistent incapability to achieve and sustain an erection suitable to support sexual intercourse [1], is a common disease and a significant contributor to poor quality of life and psychosocial morbidity in middle-aged men [2, 3]. The incidence of ED increases with age. Several studies reported that 10% of men older than 35 years experienced ED, and this percentage increased to 75% in men aged 70 and older. Therefore, it is estimated that there are more than 150 million men suffering from ED worldwide [4–6].

ED can be induced by various etiologies, including arterial, neurogenic, hormonal, cavernosal, iatrogenic, and psychogenic causes; nevertheless, the pathogenesis of ED is not clearly elucidated. Vascular endothelial dysfunction, including coronary artery disease (CAD), appears to play a significant role in the development of ED [7–9]. The possibility that CAD and ED share a common pathogenesis is no garnering substantial attentions.

Based on several systemic reviews and meta-analysis, ED and CAD share risk factors, including diabetes, tobacco abuse, alcohol abuse, hypertension, hypercholesterolemia, advanced age, obesity, and low testosterone [10, 11]. Generally, the onset of ED predates manifestations of CAD by 5 years, and 50–70% of the patients of manifesting CAD have varying degrees of ED [12–15]. Montorsi et al. reported that the severity of the ED correlates with the severity of the CAD [16]. Therefore, ED is consider more than merely sexual dysfunction, rather, it is considered a warning symptom and an independent predictor of CAD [17–19].

The internal pudendal artery (IPA), a branch of the internal iliac artery, is a major source of arterial blood flow to the penis. Several angiographic studies performed in men with ED demonstrated that vascular narrowing caused by atherosclerotic disease in IPAs, iliac arteries, and cavernous arteries is one of the mechanisms contributing to ED. In particular, the IPA appears to be closely related to the occurrence of ED [20–22]. Nevertheless, the correlation between the degree of ED and the presence and extent of IPA stenosis has not been well established.

Therefore, we evaluated the presence and extent of ED using the IIEF questionnaire in patients with suspected CAD and conducted angiography of the internal pudendal arteries to determine the correlation between the severity of ED and IPA stenosis.

## Materials and methods

This was a prospective study conducted in Daejeon St. Mary’s Hospital, the Catholic University of Korea. This study was approved by the institutional review board (IRB) of the Catholic University of Korea, Daejeon St. Mary’s Hospital (Approval Number: DC13QISI0048) and was conducted according to the Declaration of Helsinki. The patients gave written informed consent.

### 1) Study population

Patients who were expected to have ≥50% stenosis in at least one coronary artery could participate in this study based on the effort angina symptoms consistent with coronary computed tomography or evidence of exercise induced ischemia by treadmill or cardiac perfusion testing.

**Fig 1:**
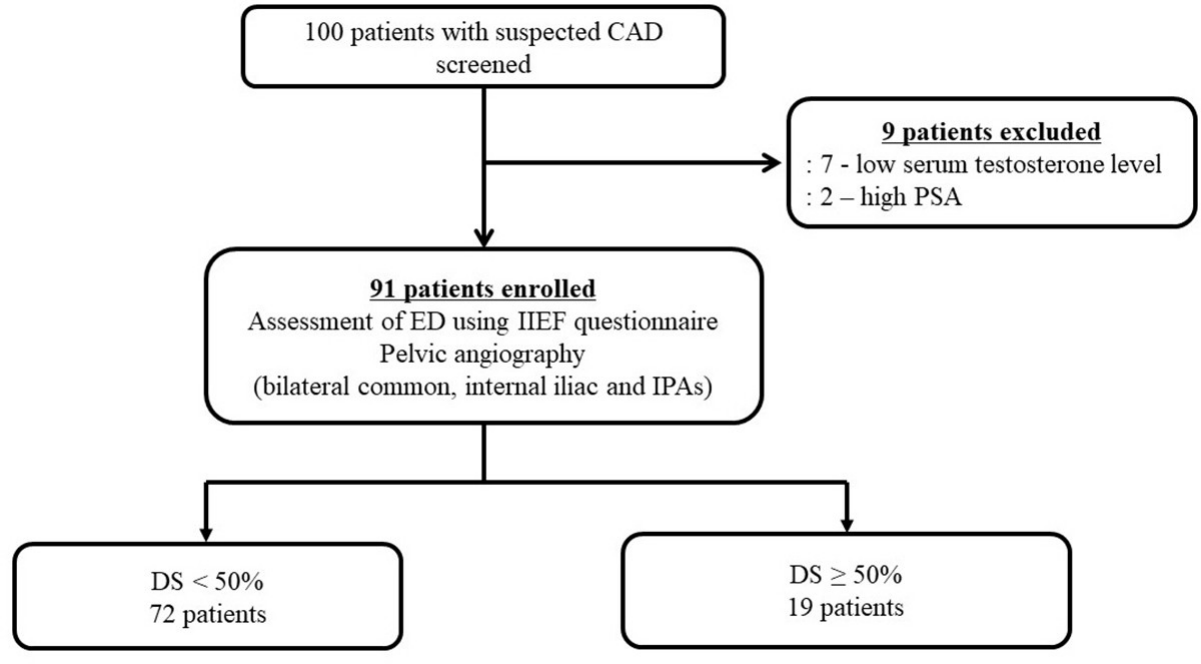
Illustration of study progress. CAD, coronary artery disease; PSA, prostate specific antigen; ED, erectile dysfunction; IIEF, international index of erectile function; IPAs, internal pudendal arteries; DS, diameter stenosis.

Subjects were excluded if they had one or more of the following characteristics: serum creatinine levels >2.5 mg/dL; cardiogenic shock; acute myocardial infarction within one month; malignancy; autoimmune disease; recent infectious disease; treatment with anti-depressive and anti-anxiety medications; current or past mood disorders, panic disorder with and without agoraphobia, social phobia, or generalized anxiety disorder; prior penile trauma; prior radical prostatectomy; low serum testosterone level; high PSA levels; prior history of pelvic radiation therapy; and alcohol or substance abuse and dependence. Study progress is illustrated in Fig 1.

A total of 91 patients were enrolled in this study. All patients underwent a baseline study examination that included a comprehensive health interview, medical history questionnaire, psychosocial questionnaire, blood sample collection, and echocardiography. To assess for the presence of depressive symptoms within the past month, a trained research assistant administered the 21-item Beck Depression Inventory II (BDI-II). Demographic data and medical history were obtained by self-report questionnaire. Age, medical history, family history of cardiovascular disease and smoking were included in the questionnaire. Complete blood count, creatinine, uric acid, fasting glucose, high-density lipoprotein cholesterol, low-density lipoprotein cholesterol, total cholesterol, triglyceride, testosterone, and hemoglobin A1c were all measured from venous blood sample, after a 12-hour fast.

### 2) Assessment of erectile dysfunction

ED was assessed using the International Index of Erectile Function (IIEF), a widely used, multi-dimensional self-report instrument for the evaluation of male sexual function. a validated, self-administered questionnaire. Among five domains, including erectile function (EF), orgasmic function, sexual desire, intercourse satisfaction, and overall satisfaction, EF domain score was used to diagnose and assess the severity of ED. ED was classified as follows: severe (EF score = 1–10); moderate (EF score = 11–16); mild-moderate (EF score = 17–21); mild (EF score = 22–25); and no ED (EF score = 26–30) [23].

### 3) Assessment of stenosis of internal pudendal artery

Vascular access was performed through the radial artery. We first performed the coronary angiography (CAG), and a lower abdominal aortogram was performed with iliofemoral run-off using a 150-cm long catheter (JSM Angio Catheter; JUNGSUNG MEDICAL Inc., Seoul, Korea). Bilateral iliac arteries and IPAs were then selected using hydrophilic wire. The IPA was selected at a posterior division of the distal internal iliac artery (arising after the origins of superior and inferior gluteal arteries), with the take off best seen in the ipsilateral cranial view. Intra-arterial nitroglycerin was administered and angiograms were taken in at least two views to visualize the entire length of the penile arterial inflow through the internal iliac arteries and IPAs to the base of the penis. Vessel size of both common iliac arteries, the internal iliac artery, and the internal pudendal artery (proximal, mid, and distal) were assessed using quantitative coronary angiography (QCA), including a proximal and distal reference diameter, calculated interpolated normal diameter, minimal lumen diameter, percentage of diameter stenosis and lesion length [24]. We divided patients into two groups: Patients with maximum stenosis of less than 50% were allocated into Group I, while patients with more than 50% were allocated into Group II, regardless of direction and location in the bilateral common, internal iliac, and IPAs.

### 4) Statistical analysis

Continuous data were expressed as the mean ± standard deviation (SD) and categorial data were expressed as count (percent). Continuous data were analyzed using Student’s t-test or Mann-Whitney U test and categorical data were analyzed using Chi-square test or Fisher’s exact test. Age group data were analyzed using analysis of variance (ANOVA) with multiple comparisons performed using the Bonferroni post hoc test. Correlation coefficient was analyzed using Pearson’s method. Statistical analyses were performed with SAS 9.4 (SAS Institute, Cary, NC). All tests were two tailed and p < 0.05 was considered significant.

## Results

### 1) Baseline clinical characteristics

Baseline clinical characteristics in the overall cohort, Group I, and Group II are presented in Table 1. The mean age was significantly higher in Group II than in Group I. On question No. 5 in the EF domain of the IIEF questionnaire, Group II scored significantly lower than did Group I.

**Table 1:** Baseline clinical characteristics.

	Total	Group I (DS <50%)	Group II (DS ≥50%)	p-value
Age	59.2±8.1	58.3±7.9	62.7±8.1	0.035*
BMI	24.9±2.9	25.0±2.9	24.4±2.9	0.388
DM	18(19.8)	14(19.4)	4(21.1)	>0.999
HBP	46(50.5)	35(48.6)	11(57.9)	0.472
Hyperlipidemia	9(9.9)	7(9.7)	2(10.5)	>0.999
Smoking	37(40.7)	27(37.5)	10(52.6)	0.232
Previous CVA	3(3.3)	2(2.8)	1(5.3)	0.509
Previous MI	25(27.5)	22(30.6)	3(15.8)	0.200
Previous PCI	44(48.4)	34(47.2)	10(52.6)	0.675
**Medication history**				
Aspirin	57(62.6)	46(63.9)	11(57.9)	0.631
Statin	55(60.4)	45(62.5)	10(52.6)	0.434
**Hormonal status**				
PSA	0.8±0.7	0.8±0.6	1.0±0.8	0.175
Testosterone	4.2±1.5	4.2±1.5	4.1±1.7	0.724
Prolactin	9.2±6.5	8.9±5.2	10.5±10.1	0.520
**IIEF questionnaire**				
Erectile function	14.4±6.8	14.8±6.7	12.6±7.0	0.197
Question No. 1	2.2±1.8	2.3±1.7	1.8±1.9	0.264
Question No. 2	2.3±1.7	2.4±1.7	1.8±1.9	0.238
Question No. 3	1.9±1.7	1.9±1.7	1.8±1.9	0.749
Question No. 4	1.8±1.6	1.9±1.5	1.7±1.8	0.737
Question No. 5	2.7±2.0	3.0±2.0	1.6±2.0	0.007*
Question No. 15	3.4±1.1	3.3±1.1	3.8±1.4	0.109
Orgasmic function	3.0±2.7	3.2±2.7	2.5±2.8	0.363
Sexual desire	5.9±2.0	6.0±2.0	5.7±2.1	0.619
Intercourse satisfaction	4.4±3.5	4.7±3.4	3.6±3.7	0.228
Overall satisfaction	6.6±2.5	6.4±2.4	7.3±3.0	0.159

BMI, body mass index; DM, diabetes mellitus; HBP, high blood pressure; Fx CAD, family history of coronary artery disease; CVA, cerebrovascular accident; MI, myocardial infarction; PCI, percutaneous coronary intervention; PSA, prostate specific antigen; DS, diameter stenosis of IPA

* Statistically significant difference between Group I and Group II

### 2) Assessment of erectile function

**Fig 2:**
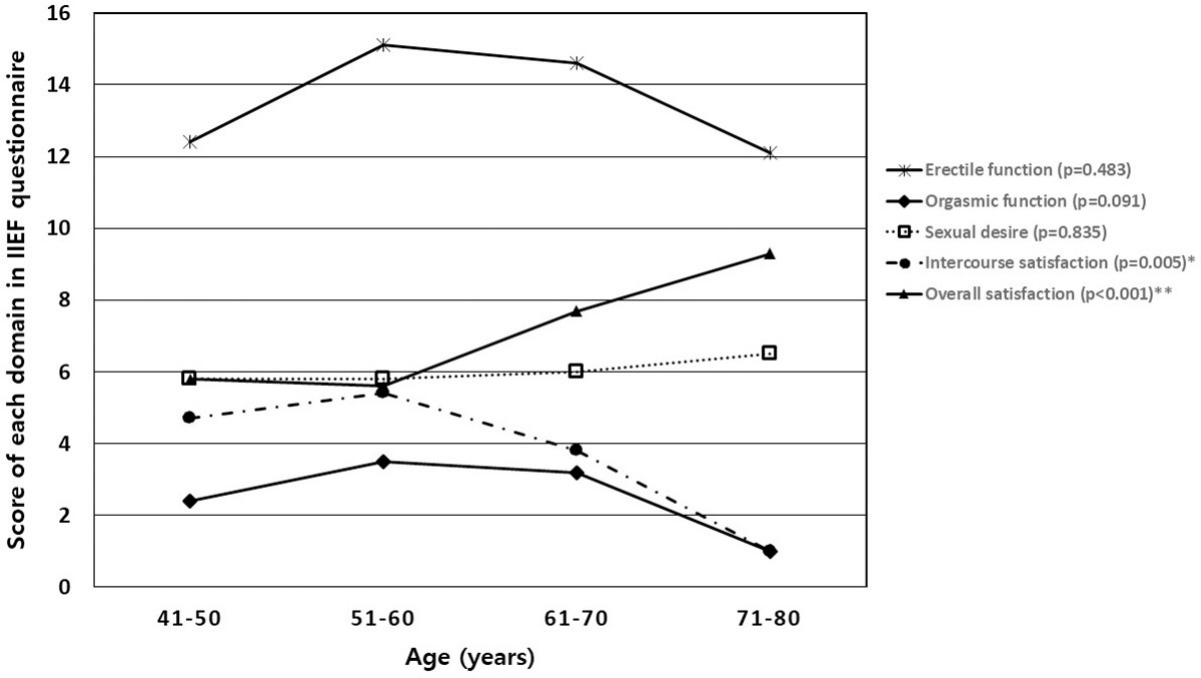
Changes of each domain in IIEF questionnaire according to the age increase. * Statistically significant decrease according to the age increase ** Statistically significant increase according to the age increase.

Each domain of the IIEF questionnaire according to increasing age showed the following pattern (Fig 2).

Among five domains of IIEF questionnaire, the intercourse satisfaction domain showed a significant decrease with increased age and the overall satisfaction domain showed a significant increase with increasing age. Nevertheless, erectile function, orgasmic function, and sexual desire did not show any significant changes as age increased.

**Fig 3:**
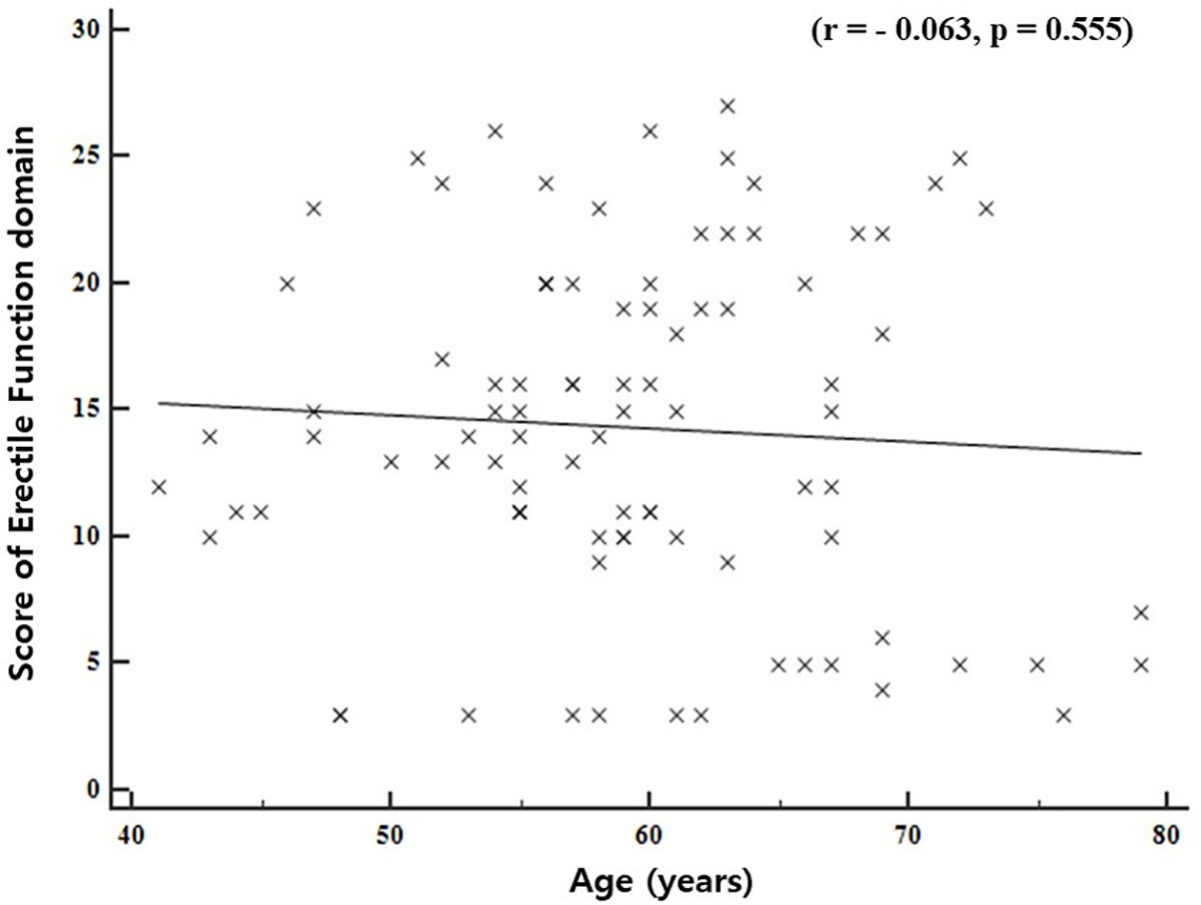
The distribution of patients by the age and the score of erectile function domain in IIEF questionnaire. r, correlation coefficient; p, p-value.

In the total of 91 patients, 88 (96.70%) were diagnosed with ED because they scored less than 25 points in the EF domain on the IIEF questionnaire. The distribution by age and severity of ED is displayed in Fig 3. There was no significant correlation between increased age and the severity of ED (r = - 0.063, p = 0.555).

### 3) Assessment of stenosis in the common iliac, internal iliac, and internal pudendal arteries

**Fig 4:**
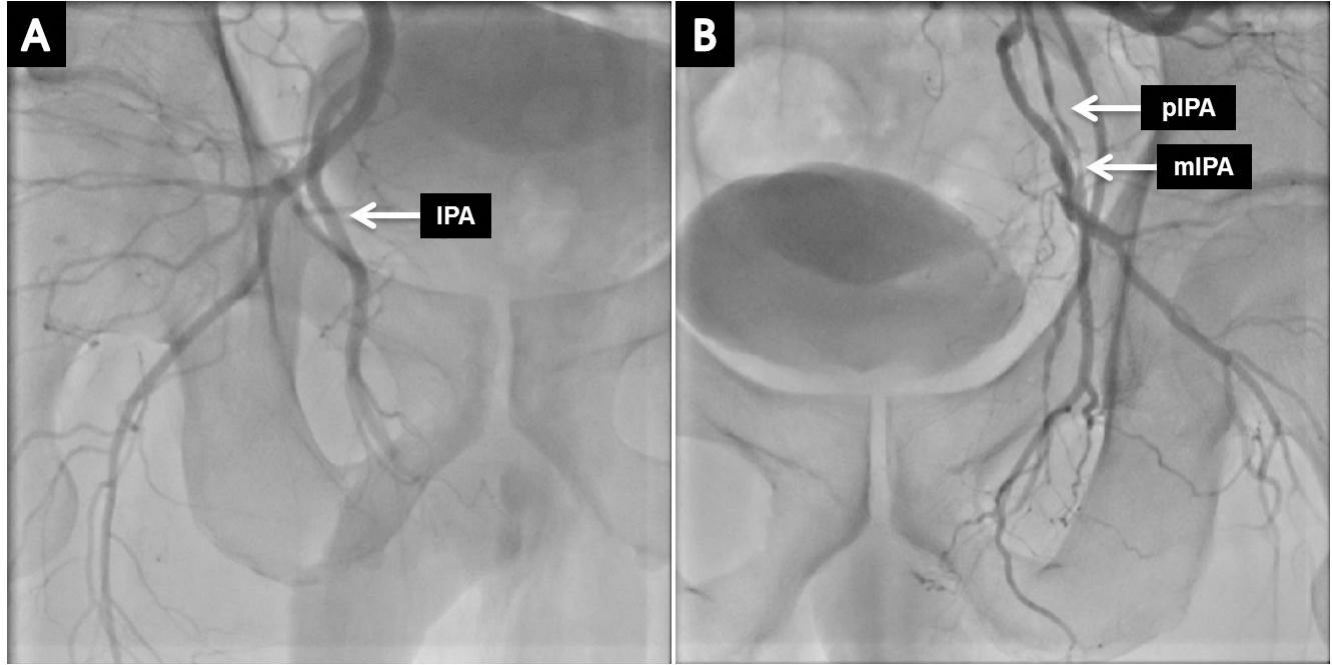
Angiographic findings of internal pudendal arteries. A: Normal Right IPA angiographic finding. B: Left proximal and middle IPA stenosis. The diameter stenosis of IPA measured by QCA method was found to be 68%(pIPA) and 69%(mIPA). IPA, internal pudendal artery; pIPA, proximal internal pudendal artery; mIPA, middle internal pudendal artery; QCA method, quantitative coronary angiographic method.

Of the total 91 patients, 26 had at least one stenotic lesion in the bilateral common, internal iliac, or IPAs, and 65 patients had no stenosis (Fig 4).

The number and extent of stenosis were similarly identified in both sides regardless of location and region. There were 15 stenotic lesions of less than 50% found in right side, while 19 were found in left side. Also, there were 22 stenotic lesions of more than 50% found in right side, while 19 were found in left side. Number of stenotic lesions of more than 50% in right and left IPAs were 18 and 15 respectively.

The reference diameter and the mean diameter of the stenotic lesion for proximal, middle, and distal parts of the IPAs through angiography were displayed in Table 2. There were 72 patients in Group I (DS <50%) and 19 in Group II (DS ≥50%). The mean extents of stenosis in each group were 4.0±12.6% and 70.0±14.8%, respectively.

**Table 2:** Reference diameter and mean diameter of stenotic lesions in bilateral internal pudendal arteries.

		Reference diameter (mm)	Mean diameter in DS < 50% (mm)	Mean diameter in DS ≥ 50% (mm)	p-value
IPA proximal	Rt	2.6 ± 0.6	2. 6± 0.5	2.3 ± 1.2	0.457
	Lt	2.7 ± 0.7	2.7 ± 0.6	2.3 ± 1.0	0.089
IPA middle	Rt	2.1 ± 0.5	2.1 ± 0.5	1.5 ± 0.9	0.018*
	Lt	2.0 ± 0.5	2.0 ± 0.5	1.5 ± 0.8	0.026*
IPA distal	Rt	1.3 ± 0.4	1.3 ± 0.4	1.1 ± 0.8	0.266
	Lt	1.2 ± 0.5	1.2 ± 0.5	1.0 ± 0.4	0.182

Data are expressed as mean ± standard deviation.

DS, diameter stenosis; IPA, internal pudendal artery.

* Statistically significant different

### 4) Correlation between severity of ED and extent of stenosis in IPAs

A total 62/65 patients who had no stenotic lesion in Group I were diagnosed with ED. Group II had a significantly higher mean age than did Group I. However, in all domains of the IIEF questionnaire, there were no significant differences between Groups.

**Fig 5:**
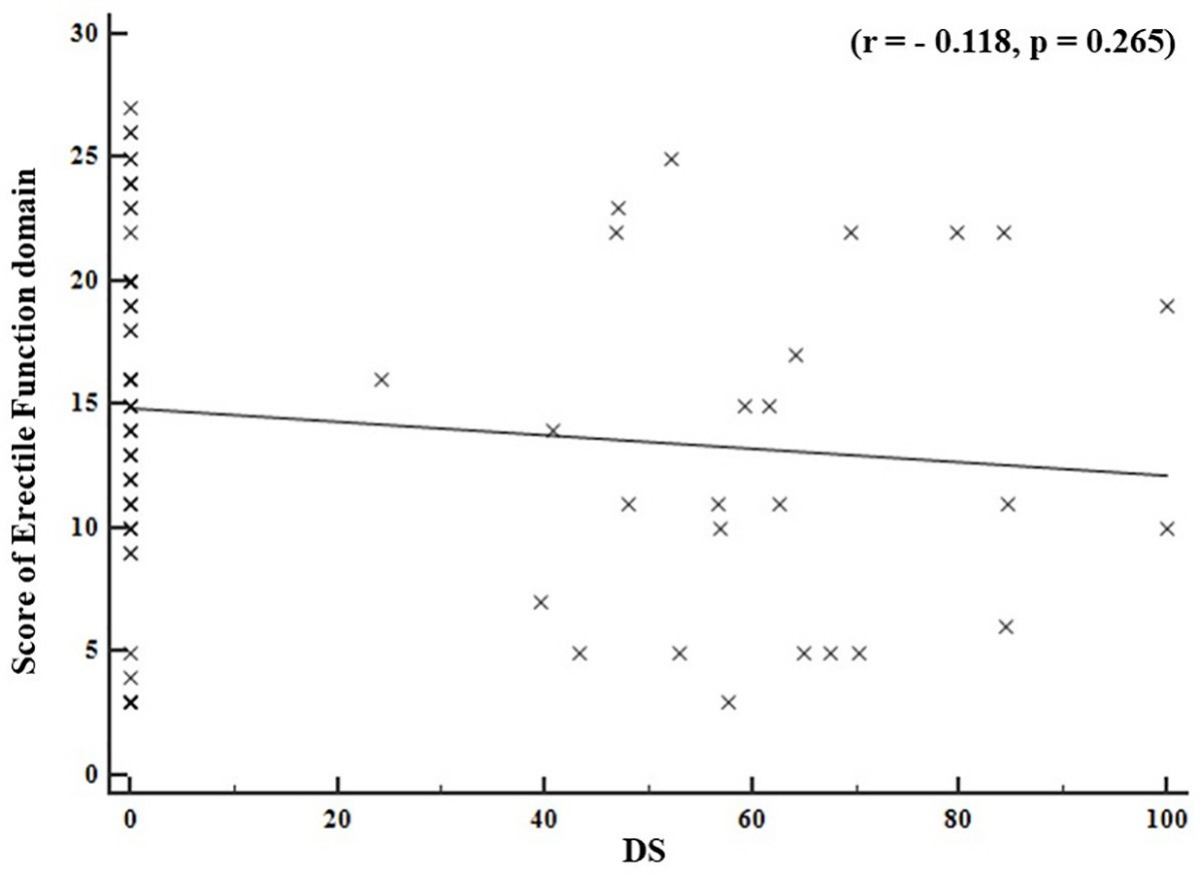
The distribution of patients according to score of EF domain in IIEF questionnaire and the extent of stenosis of IPAs. EF, erectile function; IIEF, international index of erectile function; IPAs, internal pudendal arteries, DS, diameter stenosis; r, correlation coefficient; p, p-value.

The distribution according to the severity of ED (score of EF domain on the IIEF questionnaire) and the extent of IPAs stenosis in both groups is shown in Fig 5. There was no significant correlation between the extent of stenosis of IPAs and severity of ED (r = -0.118, p = 0.265).

## Discussion

The main findings of the present study were as follows: (1) Most patients with suspected CAD were identified as having ED (88/91, 96.70%); (2) There was no clear correlation between increased age and severity of ED; (3) The extent of stenosis in IPAs increase with age; (4) Despite the absence of arterial stenosis, there were several patients diagnosed with ED; (5) There was no correlation between the extent of stenosis and the severity of ED.

Ilio-pudendal-penile arteries are associated with erectile function. Among these arteries, the IPA is the main source of blood flow to the corpora of the penis and appears to have a crucial role in erectile function. Recently, Rogers et al.[20] reported for the first time that CAD and IPA disease are closely related by showing angiographic results in patients with ED. They conducted a first-in-man ZEN (Zotarolimus-Eluting Peripheral Stent System for the Treatment of Erectile Dysfunction in Males with Suboptimal Response to PDE5 Inhibitors) study in patients with unilateral or bilateral focal IPS stenosis [21]. Zaki et al.[22] reported that significant internal iliac artery and IPA disease was found in 20% and 36.7% of 30 diabetic patients with ED and CAD, respectively.

Dose the severity of ED correlate with the extent of IPA stenosis just as the severity of ED correlates with the severity of the CAD [16]? Interestingly, we showed that the extent of IPA stenosis did not correlate with the severity of ED, and even without stenosis, ED was diagnosed. We believe we achieved this result was because ED is caused not by a reduced blood supply due to the formation of atherosclerotic plaques, but rather by vascular endothelial dysfunction prior to the formation of atherosclerotic plaques. Furthermore, we believe that the formation of atherosclerotic plaques suggests that arteries have reached a stage of dysfunction with structural changes beyond the reversible stage of dysfunction during the course of development of ED, not necessarily reflecting the extent of ED.

It is well known that sufficient arterial inflow to the penis, relaxation of cavernosal smooth muscle, and obstruction of venous outflow from the penis are essential to maintain erectile function. And several endothelium-derived factors, especially nitric oxide (NO), are involved in erection process.

The vascular endothelium plays a crucial role in the maintenance of vascular homeostasis by maintaining a delicate balance between vasodilatation and vasoconstriction [25]. Vasodilatation is mediated by factors such as NO, endothelium-derived hyperpolarizing factor (EDHF), and prostacyclin, while vasoconstriction is mediated by factors such as endothelin-1 (ET-1), angiotensin II, thromboxane A2, and prostaglandin H2 [26]. Among these endothelium-derived factors, NO is the most potent endogenous vasodilator [27] and plays a significant role in maintaining the homeostasis of the vascular wall by inhibiting platelet aggregation, inflammation, oxidative stress, vascular smooth muscle cell migration and proliferation, and leukocyte adhesion [28, 29].

Prolonged exposure to risk factors of ED can ultimately exhaust the protective effect of endogenous anti-inflammatory systems within endothelial cells [30]. This exposure induces vascular endothelial dysfunction associated with decreased NO bioavailability by impaired NO production in the endothelium or inactivation of NO by ROS [29]. This vascular endothelial dysfunction precedes the development of atherosclerotic lesions [31] and causes impairment of endothelium-dependent vasodilation and cavernous smooth muscle relaxation. Therefore, ED could occur without the formation of atherosclerotic lesions. We believe that angiographically-negative patients with ED are at this stage. Endothelial cells can lose integrity, progress to senescence, and detach into the circulation [30]. Environmental change by endothelial dysfunction leads to leukocyte adhesion and inflammation, lipid deposition, vascular smooth muscle cell proliferation, vasoconstriction, platelet aggregation, and thrombosis. As a consequence, atherosclerosis that forms intraluminal plaques [29]. We believe that patients with ED and stenosis of IPA are at this stage.

Previous studies have shown that ED is caused by vascular endothelial dysfunction [7–9] and that stenosis of arteries is a series of processes cause by endothelial cell dysfunction [29–31]. Therefore, we believe that ED can occur regardless of the presence of IPA stenosis, and the extent of IPA stenosis cannot reflect the severity of ED except when in the context of obvious stenosis of the IPA, causing significant blood flow reduction. We also believe this explains why we found no relationship between the extent of IPA stenosis and the severity of ED. We further suggest that ED without IPA stenosis can be considered a stage that can be re-routed to the previous state using treatments or medications that enhance the function of vascular endothelial cells or reduce exposure to risk factors. ED with IPA stenosis should be considered a stage in which structural change cannot be restored by the preceding methods and, in severe stenosis of IPA, active treatments such as endovascular intervention with angioplasty and DES placement are required.

There are several limitations in our study. First, it was a single-center study with relatively small sample size, and therefore may be subject to selection bias. For this reason, we instituted strict inclusion and exclusion criteria. In previous reports, Roger et al. performed IPA angiography in 10 patients with ED poorly responsive to PDE5i who were scheduled to undergo cardiac catheterization [20] and conducted ZEN in 30 patients with unilateral or bilateral focal IPS stenosis [21]. Zaki et al.[22] performed internal iliac and IPA angiography in 30 diabetic patients with ED undergoing elective coronary catheterization. Attempts at single IPA angiography to check for the presence and extent of stenosis can generate ethically significant problems without evidently severe IPA stenosis being confirmed. It is difficult to recruit patients for such a study because IPA angiography can be only performed in patients who were scheduled to undergo cardiac angiography and consented to the procedure. We believe that results of our study may be of academic significance because the 91 patients in our study constitutes a larger number than those of previous studies and multi-centre studies are needed to validate our findings in the future.

Second, the current study did not consider vascular endothelial dysfunction in the patients. Various non-invasive methods have been developed to assess vascular endothelial function, including flow-mediated dilation of brachial artery and reactive hyperemia [32], low-flow mediated constriction, endothelial peripheral arterial tonometry, and venous occlusion plethysmography [29]. Several circulating laboratory markers have been used as indicators of vascular endothelial dysfunction, including E-selectin, ICAM-1, VCAM-1, interleukin-1, tumor necrosis factor-α, interferon-γ, monocyte chemoattractant protein-1, von Willebrand factor, tissue plasminogen activator, plasminogen activator inhibitor-1, microalbuminuria, and tests of apoptosis [25]. Nevertheless, these methods and markers have several weaknesses in terms of assessing vascular endothelial function in clinical practice, including non-specific character, difficulty in standardization, and low reproducibility [29]. The development of non-invasive, reproducible, and standardized methods or makers to assess vascular endothelial function in the future will be necessary, to provide an opportunity to clarify the relationship between vascular endothelial dysfunction and ED.

## Conclusion

The development and progression of ED is a series of processes that begins with repeated exposure to risk factors, inducing vascular endothelial dysfunction. ED can occur without stenosis of the IPAs and the extent of IPAs stenosis dose not reflect the severity of ED, except when there is substantial stenosis of the IPA causing serious blood flow reduction. We believe that the development of noninvasive, reproducible, inexpensive, and standardized methods or markers to evaluate endothelial dysfunction in clinical practice will be necessary, to provide important criteria for understanding, treating, and preventing ED.
